# miR-382 Contributes to Renal Tubulointerstitial Fibrosis by Downregulating HSPD1

**DOI:** 10.1155/2017/4708516

**Published:** 2017-06-07

**Authors:** Yi Fang, Ting Xie, Ning Xue, Qing Kuang, Zheng Wei, Mingyu Liang, Xiaoqiang Ding

**Affiliations:** ^1^Department of Nephrology, Zhongshan Hospital, Fudan University, Shanghai, China; ^2^Shanghai Institute of Kidney and Dialysis, Shanghai, China; ^3^Shanghai Key Laboratory of Kidney and Blood Purification, Shanghai, China; ^4^Department of Hematology, Zhongshan Hospital, Fudan University, Shanghai, China; ^5^Department of Physiology, Medical College of Wisconsin, Milwaukee, WI, USA

## Abstract

Redox imbalance plays an important role in the pathogenesis of CKD progression. Previously, we demonstrated that microRNA-382 (miR-382) contributed to TGF-*β*1-induced loss of epithelial polarity in human kidney epithelial cells, but its role in the development of renal tubulointerstitial fibrosis remains unknown. In this study, we found that with 7 days of unilateral ureteral obstruction (UUO) in mice, the abundance of miR-382 in the obstructed kidney was significantly increased. Meanwhile, the protein expression of heat shock protein 60 (HSPD1), a predicted target of miR-382, was reduced after 7 days of UUO. Expression of 3-nitrotyrosine (3-NT) was upregulated, but expression of thioredoxin (Trx) was downregulated. Anti-miR-382 treatment suppressed the upregulation of miR-382, attenuated renal interstitial fibrosis in the obstructed kidney, and reversed the downregulation of HSPD1/Trx and upregulation of 3-NT after UUO. Furthermore, in vitro study revealed that overexpression of HSPD1 significantly restored Trx expression and reversed TGF-*β*1-induced loss of E-cadherin, while in vivo study found that direct siRNA-mediated suppression of HSPD1 in the UUO kidney promoted oxidative stress despite miR-382 blockade. Our clinical data showed that upregulation of miR-382/3-NT and downregulation of HSPD1/Trx were also observed in IgA nephropathy patients with renal interstitial fibrosis. These data supported a novel mechanism in which miR-382 targets HSPD1 and contributes to the redox imbalance in the development of renal fibrosis.

## 1. Introduction

Renal tubulointerstitial fibrosis (TIF) is a prominent pathological feature of chronic kidney disease (CKD) and the pathway leading to end-stage renal disease. It involves an excess accumulation of extracellular matrix proteins in the renal interstitium and is associated with inflammatory cell infiltration, tubular cell loss, and fibroblast accumulation [1–4]. In our previous study, we found that microRNA-382 (miR-382) contributed to TGF-*β*1-induced loss of epithelial characteristics in cultured human kidney epithelial (HK2) cells [5]. Proteomic and bioinformatic analyses revealed that heat shock protein 60 (HSPD60, HSPD1) was a target gene of miR-382 [5]. HSPD1 is a key protein that maintains mitochondrial integrity and cellular activity, thereby protecting the cell from oxidative stress by assisting in mitochondrial protein folding and inhibiting apoptosis [6–9]. Since enhanced oxidative stress or redox imbalance has been proved to correlate with renal dysfunction [10, 11], miR-382 or HSPD1 might potentially serve as a new target for therapy in advanced CKD. Therefore, the goals of this study mainly were to verify the complementary relationship between miR-382 and HSPD1, as well as to further explore the role of miR-382 in the development of renal tubulointerstitial fibrosis.

## 2. Materials and Methods

### 2.1. Cell Culture and Drug Treatments

Human kidney epithelial (HK2) cells were obtained from ATCC (Manassas, Virginia, USA). DMEM (Gibco), Opti-MEM I (Gibco), or other cell culture media were used according to the manufacturer's recommendations. HK2 cells at approximately 40% confluency were treated with recombinant human TGF-*β*1 (3 ng/ml) (R&D Systems) or the vehicle control in DMEM for the indicated time period. Pre-miR-382 or anti-miR-382 oligonucleotides were obtained from Ambion. HK2 cells were transfected with the oligonucleotides (100 nM) using Oligofectamine (Invitrogen).

### 2.2. Mouse Model of UUO

Laboratory animals: ICR mice (6–8 weeks, 23–26 g, male) were purchased from Shanghai SLRC Laboratory Animal Co. Ltd. (Shanghai, China). Animals were housed in temperature- and humidity-controlled cages, with free access to water and rodent food on a 12 h light/dark cycle. Experiments were completed at Fudan University, and all protocols were approved by the Institutional Animal Care and Use Committee of Fudan University, as well as adhered strictly to the NIH Guide for the Care and Use of Laboratory Animals. For mice which became severely ill or moribund during the experiment, euthanasia was performed according to euthanasia guidelines of animals issued by the Institutional Animal Care and Use Committee of Fudan University.

All surgeries were performed under intraperitoneal sodium pentobarbital anesthesia (80 mg/kg), intrarectal temperature of mice was maintained at 36.5°C–37.0°C with a heating pad during the surgeries, and efforts were made to minimize suffering and the number of mice used. Mice underwent unilateral ureteral obstruction (UUO) or sham operation of the left ureter. The obstruction was produced by ligation of the left ureter following midline laparotomy. The left ureter was isolated, but not ligated, in sham-operated controls. After UUO surgery, mice were transferred to the recovery cages, one cage each. Parameters like vital signs and time to wake up from anesthesia were monitored. Food intake was monitored every day and animals were weighed once every other day. Locked nucleic acid- (LNA-) modified anti-miR-382 oligonucleotides antiscrambled (Exiqon) were diluted in saline (5 mg/ml) and intravenously delivered via tail vein (10 mg/kg) within 30 mins prior to UUO, and the dosage was repeated 24 hours after the surgery. In vivo delivery of small interfering RNA (siRNA) designed against HSPD1 or negative control siRNA (10 *μ*g, GenePharma) was performed by retrograde injection into the ureter proximal to the ligature, immediately following occlusion of the ureter. Mice were sacrificed by cervical dislocation 7 days after left ureteral obstruction. The kidneys were collected for evaluating the abundance of miR-382, HSPD1, thioredoxin (Trx), 3-nitrotyrosine (3-NT), VCAM-1, and CD3. The degree of renal fibrosis was graded according to Masson trichrome staining and Sirius red staining.

### 2.3. Patient Selection and Renal Morphologic Analysis

We studied 12 patients with primary IgA nephropathy between 2014 and 2015, and all diagnoses were confirmed by kidney biopsy at Zhongshan Hospital, Fudan University. Six patients had no tubulointerstitial fibrosis, and 6 patients exhibited moderate to severe tubulointerstitial fibrosis. Analysis of renal fibrosis was performed with 2 *μ*m paraffin-embedded sections stained by Masson trichrome and Sirius red stain. The degree of renal fibrosis was scored by an experienced pathologist. For tubulointerstitial fibrosis, 10 microscopic fields were viewed at a magnification of ×400 and scored subjectively from 0 to 100% for each patient. The degree of tubulointerstitial fibrosis was graded on a scale of 0 to 4: grade 0 had no affected area (normal); grade 1 had an affected area of less than 10%; grade 2 had an affected area of 10 to 25%; grade 3 had an affected area of 25 to 75%; grade 4 had an affected area of greater than 75% [12]. The resulting index in each slide was expressed as a mean of all scores obtained. The same method was applied in the analysis of mouse renal pathology. The study was approved by the Clinical Research Ethical Committee of Zhongshan Hospital, Fudan University. All patients provided written informed consents. Clinical data including blood pressure, serum creatinine, and 24 h urine protein tests were recorded at the time of kidney biopsy.

### 2.4. RNA Isolation

Total RNA was extracted using TRIzol (Invitrogen) from cells or tissue sections as previously described [5, 13]. Total RNA from formalin-fixed and paraffin-embedded (FFPE) tissues was extracted by RecoverALL total nucleic acid isolation kit (Ambion, Austin, Texas, USA) according to the manufacturer's protocol.

### 2.5. TaqMan Real-Time PCR

Expression levels of miR-382 were quantified by real-time reverse transcription-PCR with the TaqMan chemistry (Applied Biosystems) as previously described [5, 13]. The mRNA levels of HSPD1, *α*-SMA, E-cadherin, Vimentin, Trx, and GAPDH were quantified using SYBR Green as previously described [5, 13]. Total RNA samples were adopted in these analyses. 5S rRNA and GAPDH mRNA were used as normalization controls for miRNA and protein-coding genes, respectively. Relative changes in mRNA and miR-21 expression were determined using the 2^−ΔΔCt^ method. Relative gene levels were expressed as ratios to control. The sequences of primers are listed in Table 1.

### 2.6. Western Blot and Immunohistochemistry

The relative abundance of several proteins was analyzed using Western blot or immunohistochemistry (IHC) as previously described [5, 13]. Primary antibodies for HSPD1 (1 : 100; Enzo Life Sciences), E-cadherin (1 : 100; Abcam), Vimentin (1 : 100; Abcam), *α*-SMA (1 : 75; Sigma-Aldrich), 3-NT (1 : 100; Abcam), Trx (1 : 100; Abcam), VCAM-1 (1 : 10;0 Abcam), CD3 (1 : 50; Abcam), and Bax (1 : 100; Cell Signaling Tech) were applied according to the manufacturer's instructions. Secondary antibodies (1 : 1000; Jackson ImmunoResearch) were also applied according to the manufacturer's instructions. All immunohistochemistry staining and collagen staining were analyzed as the following steps: under a microscope (400x), we move the slices randomly and take 20–25 pictures per slice, then analyze by Image Pro-Plus 6.0 Software (Media Cybernetics, USA) to quantify the abundance of our target protein/gene. Parameter of IOD (integrated optical density) was employed to do this work. To prove our assumption with a second method, we also quantified tyrosine-nitrosylated proteins by fluorescence immunoblotting of 3-NT (Abcam; 1 : 100). Secondary antibodies were species-specific FITC-conjugated IgG (1 : 500, Invitrogen).

### 2.7. 3′-Untranslated Region (UTR) Reporter Analysis

3′-UTR reporter constructs were generated, and reporter activity was analyzed as previously described with minor modifications [5, 13]. Briefly, HeLa cells (80–90% confluency) were cotransfected with the following: a 3′-UTR reporter construct (100 ng per well), a pRL-TK internal control plasmid (50 ng per well), and control pre-miR-382 oligonucleotides (10 pmol per well, from Ambion). Cells were cotransfected using Lipofectamine 2000 (Invitrogen), following the manufacturer's protocol. Firefly and Renilla luciferase activities were measured in each well 24 h after the transfection using the Dual-Luciferase Reporter Assay System (Promega), following the manufacturer's protocol. Renilla luciferase activity was used as a normalization control for luciferase activity to measure transfection efficiency and cell density.

Site-directed mutagenesis was performed with the QuickChange II XL site-directed mutagenesis kit (Stratagene), following the manufacturer's protocol. Primers used for introducing point mutations for HSPD1 were as follows: forward primer, 5- CAAGGCAGTGTTCCTCACCAATAgaTTCAGAGAAGACAGTTG -3; reverse primer, 5- CAACTGTCTTCTCTGAAtcTATTGGTGAGGAACACTGCCTTG -3. Underlined nucleotides represent the mutations introduced, and these nucleotides are located in the core region of the predicted target site for miR-382.

### 2.8. miR-382 In Situ Hybridization

Expression of miR-382 was detected by in situ hybridization (ISH), following the protocol of “One-day microRNA ISH” suggested by Exiqon. The experimental conditions for renal tissues of IgA nephropathy patients were as follows: FFPE kidney tissues were cut into 2 *μ*m thick sections, cleared in xylenes, rehydrated using an ethanol gradient, and then exposed to a 10 min proteinase K (15 mg/ml) treatment at 37°C. The probe was diluted in hybridization buffer (60 nM, 60 *μ*l/tissue section) and preheated at 90°C for 4 min to linearize. The probe was then added to the slides and incubated for 90 min at 54°C. Sheep antidigoxigenin alkaline phosphatase (anti-DIG-AP) antibody was diluted (1 : 1000) and added to the slides, which were incubated at room temperature for 60 min. Slides were incubated in nitro blue tetrazolium/5-bromo-4-chloro-3-indolyl phosphate (NBT/BCIP; Roche) diluted with double-distilled H_2_O at 32°C, and tissues were then mounted with neutral resin.

### 2.9. Plasmid Transfection

HSPD1 human cDNA ORF clone was obtained from KeyGentec. The clone expresses HSPD1 driven by a CMV promoter and tagged with C-terminal Myc-DDK. The clone was amplified and purified as previously described [5]. HK2 cells were transfected with HSPD1 plasmid or an empty vector using Lipofectamine 2000 with a working concentration of 2 *μ*g for each 35 mm dish.

### 2.10. Measurement of Cellular ROS Level

CM-H2DCFCA, a ROS-sensitive fluorescent dye, was used to measure ROS levels, as previously described [14]. HK-2 cells were incubated in 96-well plates. 5 mM CM-H2DCFCA was added to each well. Fluorescence intensities were measured using a microplate fluorescence reader.

### 2.11. Statistical Analysis

Data were expressed as mean ± SD. Differences among three or more groups were evaluated using one-way analysis of variance (ANOVA) with Bonferroni adjustment. SPSS software (version 19.0) was used for statistical analysis. A value of *P* < 0.05 was considered to be statistically significant.

## 3. Results

### 3.1. miR-382 Contributes to the Progression of Renal Tubulointerstitial Fibrosis in UUO Mice

**Figure 1:**
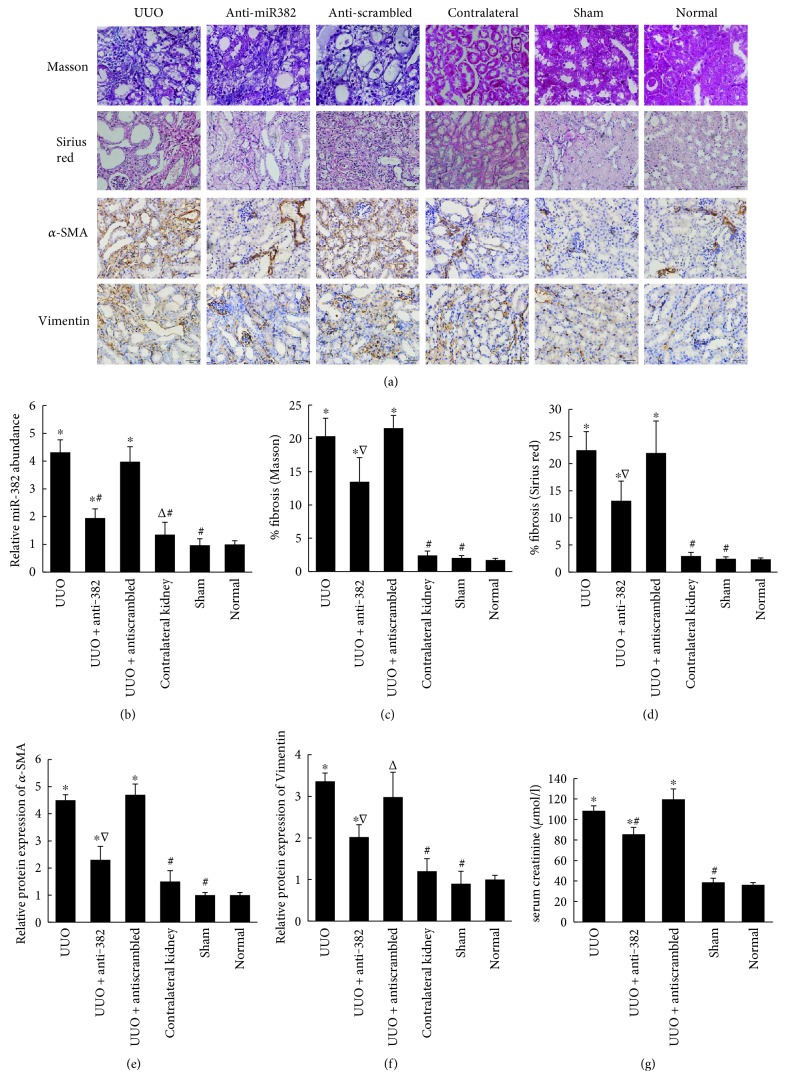
miR-382 contributed to the progression of renal tubulointerstitial fibrosis in UUO mice. (a) Staining of collagen, *α*-SMA, and Vimentin. (b) Relative abundance of miR-382. (c) The abundance of collagen was analyzed using Masson trichrome staining (Masson trichrome staining ×400, *n* = 4). (d) The abundance of collagen was analyzed using Sirius red staining (Sirius red staining ×400, *n* = 4). (e) Quantification of *α*-SMA staining. (f) Quantification of Vimentin staining. The abundance of collagen, *α*-SMA, or Vimentin was quantitatively measured using Image Pro-Plus 6.0 Software (Media Cybernetics, USA). Relative protein levels were expressed as ratios to control. (g) Serum creatinine concentration. Mice underwent unilateral ureteral obstruction or sham operation of the left ureter. Locked nucleic acid-modified anti-miR-382 oligonucleotides and antiscrambled were diluted in saline (5 mg/ml) and intravenously delivered via tail vein (10 mg/kg) within 30 min prior to UUO, and the dosage was repeated 24 hours after the surgery. ^Δ^*P* < 0.05, ^∗^*P* < 0.01 compared with the normal group; ^∇^*P* < 0.05, ^#^*P* < 0.01 compared with the UUO group. UUO indicates unilateral ureteral obstruction. Normal indicates the normal control group.

**Figure 2:**
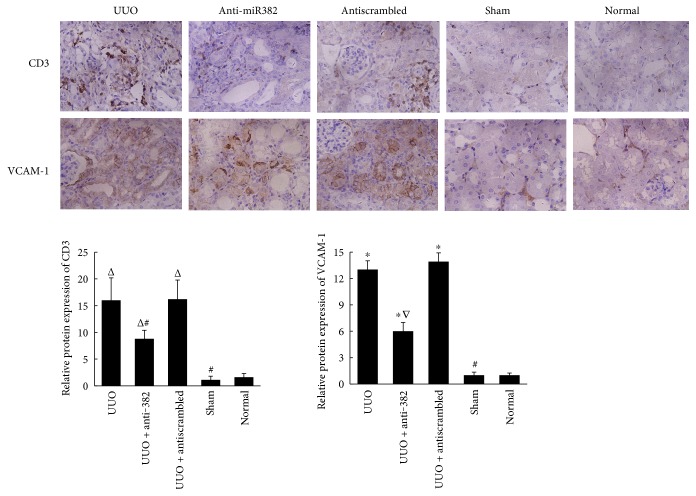
Expression of CD3 and VCAM-1 in the obstructed mouse kidneys. Treatment with 10 mg/kg anti-miR-382 reversed the upregulation of CD3 and VCAM-1. The positive stained area of VCAM-1 staining or CD3 was quantitatively measured by Image-Pro Plus, as described in Figure 1. ^Δ^*P* < 0.05, ^∗^*P* < 0.01 compared with the normal group; ^∇^*P* < 0.05, ^#^*P* < 0.01 compared with the UUO group. UUO indicates unilateral ureteral obstruction. Normal indicates the normal control group (*n* = 4).

Tubulointerstitial fibrosis developed after 7 days of UUO in the obstructed kidneys (Figure 1(a)), and the expression of miR-382 was higher in the UUO group (UUO versus control, 4.32 ± 0.45 versus 1.00 ± 0.13, resp., *P* < 0.01). Locked nucleic acid- (LNA-) modified anti-miR-382 (10 mg/kg) was delivered by tail vein injection 30 min prior to UUO, and the dosage was repeated 24 h after the surgery. In the anti-miR-382 group, the expression of miR-382 was significantly suppressed by 10 mg/kg LNA-anti-miR-382 treatment compared with the antiscrambled (10 mg/kg anti-382 versus antiscrambled, 1.95 ± 0.33 versus 3.98 ± 0.54, resp., *P* < 0.05) (Figure 1(a)). Renal histological analysis showed that blocking the expression of miR-382 could attenuate renal interstitial fibrosis (Figures 1(a), 1(c), and 1(d)) and the immunohistochemical staining indicated that the upregulation of *α*-SMA and Vimentin was partially reversed in obstructed kidneys after 10 mg/kg anti-miR-382 treatment (Figures 1(e) and 1(f)). Besides, anti-miR382 treatment also attenuated renal injury with less increased serum creatinine (Figure 1(f)) and blocked inflammatory cell infiltration in the obstructed kidney (Figure 2).

### 3.2. Identification of HSPD1 as a New Target of miR-382

**Figure 3:**
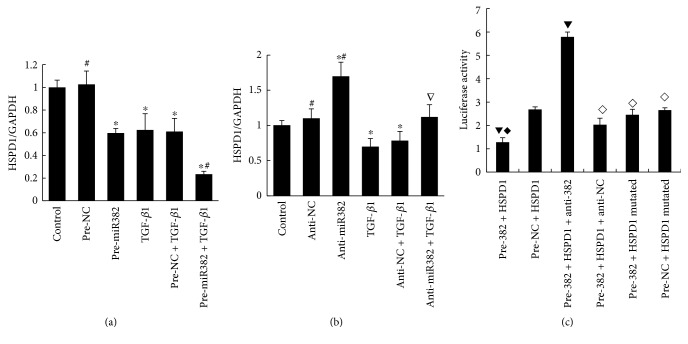
Interaction between miR-382 and HSPD1. (a) Both TGF-*β*1 and pre-miR-382 could repress the protein expression of HSPD1 in HK2 cells. (b) Anti-miR-382 treatment could partially reverse the downregulation of HSPD1 that is induced by TGF-*β*1 in HK2 cells. (c) MiR-382 interacted with the 3′-UTR of HSPD1. HeLa cells at ~80% confluency were cotransfected with a 3′-UTR segment of HSPD1 (marked with luciferase reporter gene), a pRL-TK internal control plasmid, and control pre-miR oligonucleotides or the miR-382. Luciferase activity was significantly decreased by pre-miR-382, while in the mutated group, pre-miR-382 did not have significant effect on luciferase activity. ^∗^*P* < 0.01 compared with the control group; ^∇^*P* < 0.05, ^#^*P* < 0.01 compared with the TGF-*β*1 group. ^▼^*P* < 0.05 compared with the pre-NC+HSPD1 group; ^◇^*P* < 0.05, ^◆^*P* < 0.01 compared with the pre-382 + HSPD1 + anti-382 group. NC indicates negative control. Anti-382 indicates the group treated with anti-miR-382 oligos. Pre-382 indicates the group treated with pre-miR-382 oligos. Anti-NC indicates antinegative control. Pre-NC indicates prenegative control. UUO indicates unilateral ureteral obstruction. (a, b) Western blot; (c) 3′-UTR luciferase reporter assay and site-directed mutagenesis (*n* = 5).

According to our previously published data, we found that miR-382 targeted a cluster of oxidative-related genes including HSPD1 [5]. In this study, we found that transfecting HK2 cells with pre-miR-382 significantly suppressed the protein expression of HSPD1 (pre-382 versus pre-NC, 0.60 ± 0.04 versus 1.00 ± 0.04, resp., *P* < 0.05) (Figure 3(a)). Anti-miR-382 oligo treatment inhibited the upregulation of miR-382 and reversed the decrease of HSPD1 expression induced by TGF-*β*1 in cultured HK2 cells (Figure 3(b)). A 3′-UTR segment of HSPD1 was cloned into a gene expression vector with a luciferase reporter gene. Cotransfection with pre-miR-382 to the HeLa cells reduced the luciferase activity significantly. Mutations introduced into the predicted binding site of miR-382 within the 3′-UTR of HSPD1 prevented the suppression of HSPD1 by pre-miR-382 (Figure 3(c)).

### 3.3. Overexpression of miR-382 Reduces the Antioxidant Capacity of Renal Tissues by Downregulating HSPD1

**Figure 4:**
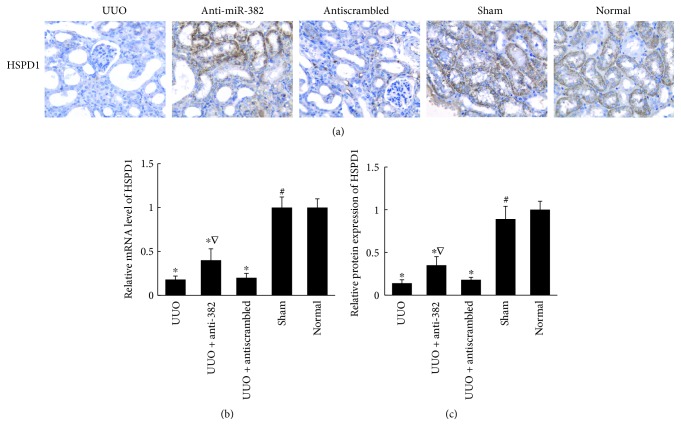
Expression of HSPD1 in obstructed mouse kidneys. (a) Immunohistochemical analysis of HSPD1 abundance in kidney specimens from mice. 10 mg/kg dosage of anti-miR-382 treatment suppressed the downregulation of HSPD1 in UUO mice. (b) Histogram represented the relative mRNA expression of HSPD1 in the mouse kidneys. (c) Quantification of HSPD1 staining. ^∗^*P* < 0.05 compared with the normal group; ^∇^*P* < 0.05, ^#^*P* < 0.01 compared with the UUO group. UUO indicates unilateral ureteral obstruction. Normal indicates the normal control group. (*n* = 4).

**Figure 5:**
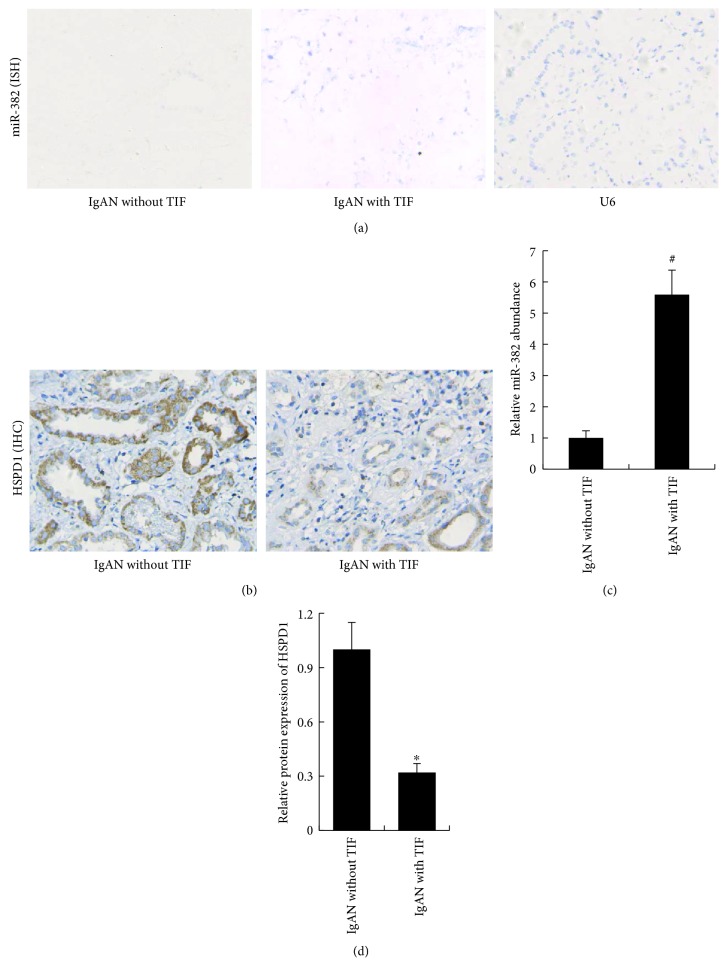
MiR-382 abundance and HSPD1 in patients with IgA nephropathy (IgAN). (a) Representative images of miR-382 expression in renal biopsy specimens from IgA nephropathy (IgAN) patients, obtained by in situ hybridization (ISH). (b) Immunohistochemical (IHC) analysis of HSPD1 abundance in renal biopsy specimens. (c) Histogram represented the TaqMan qPCR analysis of miR-382 abundance. Total RNA from formalin-fixed and paraffin-embedded tissue was extracted by using RecoverALL total nucleic acid isolation kit (Ambion, Austin, Texas, USA). (d) Histogram represented the relative protein expression of HSPD1 of renal biopsy specimens from IgAN patients. The positively stained area of HSPD1 protein was quantified as described in Figure 1. TIF indicates tubulointerstitial fibrosis. ^∗^*P* < 0.05, ^#^*P* < 0.01 compared with IgAN without TIF (IHC, *n* = 6; ISH, *n* = 6).

**Figure 6:**
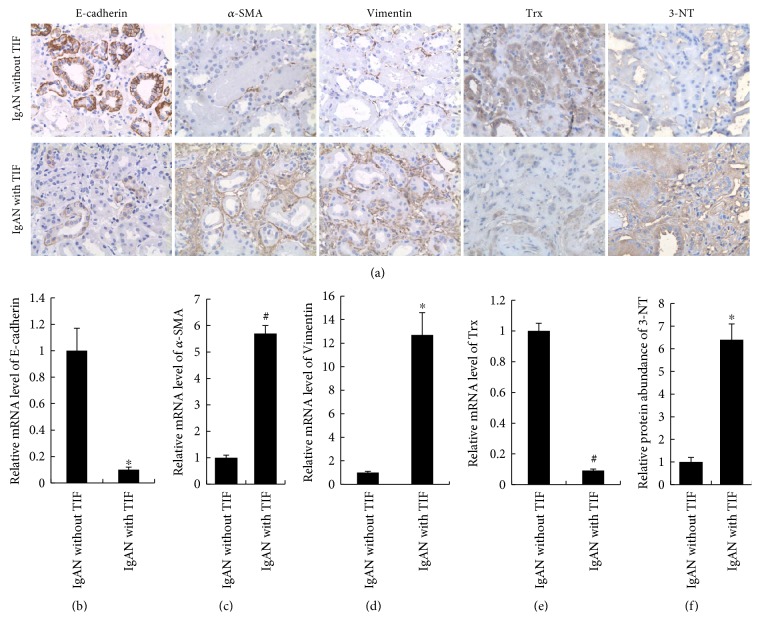
Expression of EMT markers (E-cadherin, *α*-SMA, and Vimentin) and oxidative stress-related markers (Trx, 3-NT) in patients with IgAN. (a) Representative images of protein expression of E-cadherin, *α*-SMA, Vimentin, thioredoxin (Trx), and 3-nitrotyrosine (3-NT) in renal biopsy specimens from IgA nephropathy (IgAN) patients. Immunohistochemical analysis was performed between patients with and without tubulointerstitial fibrosis (TIF). Histograms (b–e) represented the relative mRNA expression of E-cadherin, *α*-SMA, Vimentin, and Trx. (f) Histogram represents the relative protein abundance of 3-NT. The positively stained area of 3-NT protein was quantified as described in Figure 1. ^∗^*P* < 0.05, ^#^*P* < 0.01 compared with IgAN without TIF (IHC, *n* = 6; qPCR, *n* = 6).

The inverse relationship between miR-382 expression and renal expression of HSPD1 also exists in the obstructed kidneys of UUO mice as well as in patients with chronic kidney disease. There was a lower protein expression of HSPD1 after 7 days of UUO in the obstructed murine kidney (UUO versus normal, 0.14 ± 0.04 versus 1.00 ± 0.11, resp., *P* < 0.05). Anti-miR-382 treatment with a dosage of 10 mg/kg suppressed the downregulation of HSPD1 in UUO mice (Figure 4). In the clinical setting, patients with IgA nephropathy (IgAN) were identified, and cases were selected based on whether the diagnosis was minimal or substantial renal interstitial fibrosis. In IgAN patients with TIF, miR-382 abundance was significantly upregulated compared to that in IgAN patients with no TIF (5.59 ± 0.79 versus 1.00 ± 0.23, *P* < 0.01, Figure 5(c)). Comparative protein abundance of E-cadherin was suppressed (TIF versus no TIF, 0.33 ± 0.18 versus 1.00 ± 0.47, resp., *P* < 0.05), while both *α*-SMA (TIF versus no TIF, 4.29 ± 1.72 versus 1.00 ± 0.20, resp., *P* < 0.01) and Vimentin (TIF versus no TIF, 4.60 ± 1.82 versus 1.00 ± 0.26, resp., *P* < 0.05) were augmented in the TIF group (Figure 6). There also existed an inverse relationship between HSPD1 and miR-382 abundance. The protein abundance (TIF versus no TIF, 0.12 ± 0.04 versus 1.00 ± 0.18, resp., *P* < 0.05) of HSPD1 was significantly reduced in patients with TIF (Figure 5(d)).

**Figure 7:**
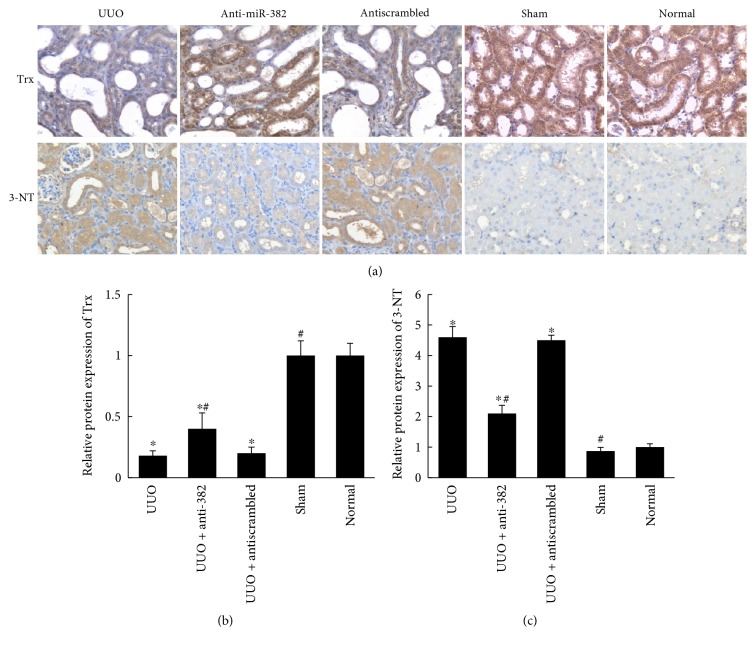
Expression of oxidative stress-related markers (Trx, 3-NT) in obstructed mouse kidneys. Treatment with 10 mg/kg anti-miR-382 reversed the downregulation of thioredoxin (Trx) and upregulation of 3-nitrotyrosine (3-NT) in UUO mice. (a) Representative images of Trx and 3-NT expression in mouse kidneys from all treatment groups. (b, c) Histogram represents the quantification of Trx and 3-NT staining in mouse kidneys. The positively stained area of 3-NT or Trx was quantified as described in Figure 1. ^∗^*P* < 0.05 compared with the normal group; ^#^*P* < 0.05 compared with the UUO group. UUO indicates unilateral ureteral obstruction. Normal indicates normal control (*n* = 4).

Immunohistochemical staining analysis showed that the comparative protein expression of thioredoxin (Trx), a marker of antioxidant capacity, was reduced in the obstructed kidneys (Figure 7). In contrast, the protein expression of 3-nitrotyrosine (3-NT), a marker of oxidative stress, was upregulated (Figure 7). LNA-anti-miR-382 treatment (10 mg/kg) significantly reversed the downregulation of Trx and suppressed the expression of 3-NT in mice subjected to UUO (10 mg/kg anti-382 group versus anti-NC group, Trx: 0.43 ± 0.13 versus 0.20 ± 0.06, *P* < 0.05; 3-NT: 2.11 ± 0.27 versus 4.52 ± 0.16, *P* < 0.05). Similar profiles of Trx and 3-NT expression were also detected in the renal biopsy tissues of IgAN patients (TIF versus no TIF, Trx: 0.35 ± 0.09 versus 1.00 ± 0.15, *P* < 0.05; 3-NT: 1.00 ± 0.24 versus 6.43 ± 0.69, *P* < 0.05) (Figure 6).

### 3.4. Overexpression of HSPD1 Restores Renal Antioxidant Capacity and Attenuates TGF-*β*1-Induced Loss of Cell Polarity In Vitro

**Figure 8:**
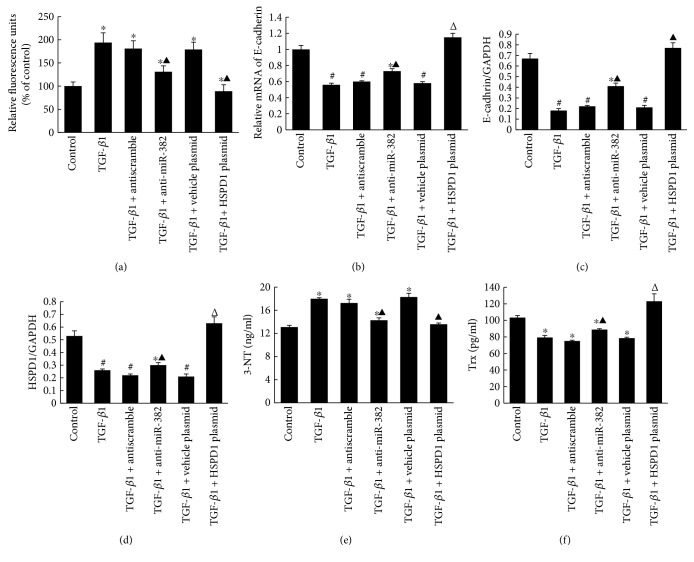
Overexpression of HSPD1 restored renal antioxidant capacity and attenuated TGF-*β*1-induced loss of cell polarity in vitro. HK2 cells were not transfected or were transfected with Myc-DDK-tagged plasmid (pHSPD1) or an empty vector. Cells without plasmid transfection were transfected with LNA-anti-miR382/anti-scramble oligos. (a) Effects of anti-382 treatment or HSPD1 overexpression on ROS levels in HK2 cells under TGF-*β*1 exposure. Fluorescence intensity was measured at an excitation wavelength of 485 nm and an emission wavelength of 535 nm. (b) Relative mRNA level of E-cadherin. (c, d) Relative protein abundance of E-cadherin and HSPD1, examined by Western blot analysis. (e) ELISA assay of 3-NT (Abcam) with the cell homogenate. (f) ELISA assay of Trx (BioVendor R&D) with the cell homogenate. 3-NT indicates 3-nitrotyrosine; Trx indicates thioredoxin. ^∗^*P* < 0.05, ^#^*P* < 0.01 compared with the control group; ^▲^*P* < 0.05, ^Δ^*P* < 0.01 compared with the TGF-*β*1 group (*n* = 5).

The cellular ROS production induced by TGF-*β*1 in the TGF-*β*1+HSPD1 plasmid group was significantly lower than that in the TGF-*β*1+vehicle plasmid group (relative fluorescence units, 89.67 ± 14.15 versus 179.55 ± 16.32, *P* < 0.05) (Figure 8(a)). HK2 cells were quite resistant to TGF-*β*1 exposure with pretreatment of HSPD1 plasmid transfection, due to the similar levels both mRNA and protein of E-cadherin maintained compared to those of the control groups (Figures 8(b) and 8(c)). ELISA assay of the cell homogenate revealed that TGF-*β*1-induced downregulation of Trx was completely restored by HSPD1 transfection (TGF-*β*1 + HSPD1 plasmid group versus TGF-*β*1 + vehicle group, 122.99 ± 9.08 pg/ml versus 78.43 ± 1.21 pg/ml, resp., *P* < 0.01; TGF-*β*1 + HSPD1 plasmid group versus control group, 122.99 ± 9.08 pg/ml versus 103.24 ± 2.58 pg/ml, resp., *P* > 0.05), while the protein expression of 3-NT in HK2 cells, as a biomarker of oxidative stress and inadequate cellular antioxidant defenses, was attenuated after HSPD1 plasmid transfection (TGF-*β*1 + HSPD1 plasmid group versus TGF-*β*1 + vehicle plasmid group, 13.57 ± 0.24 ng/ml versus 18.28 ± 0.66 ng/ml, resp., *P* < 0.01; TGF-*β*1 + HSPD1 plasmid group versus control group, 13.57 ± 0.24 ng/ml versus 13.08 ± 0.23 ng/ml, resp., *P* > 0.05) (Figures 8(e) and 8(f)). The above results suggested that overexpression of HSPD1 might protect renal epithelial cells from oxidative stress-induced loss of cell polarity.

### 3.5. Renal HSPD1 Mediates the Protective Effects of miR-382 Blockade against Renal Tubulointerstitial Fibrosis

**Figure 9:**
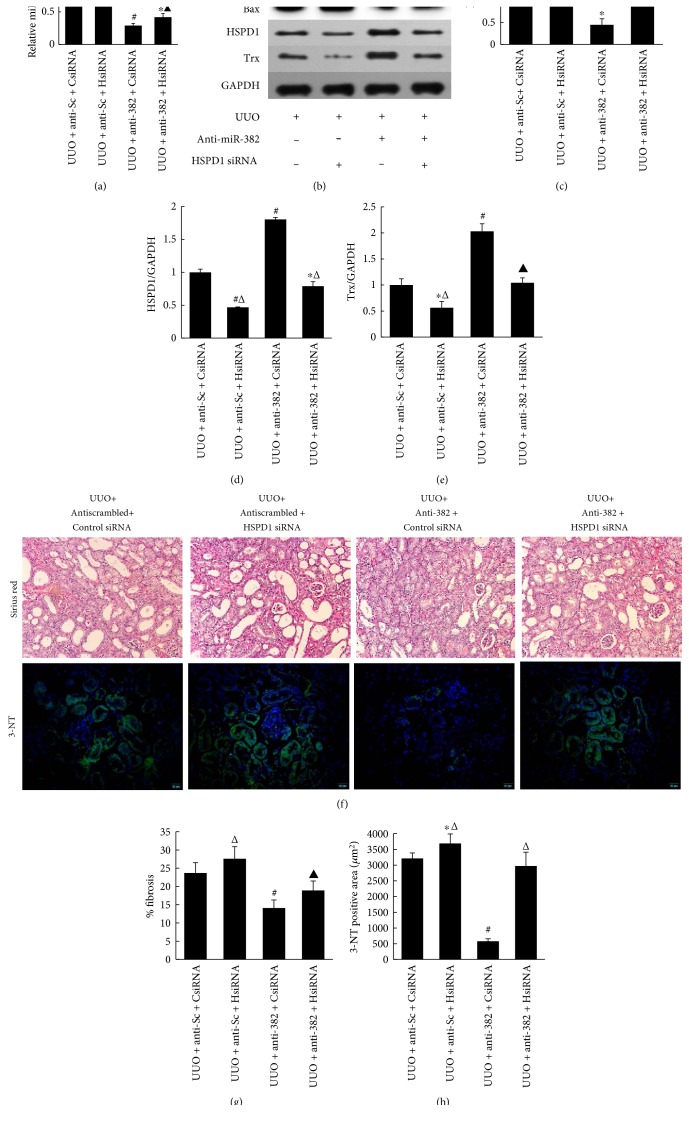
Direct siRNA-mediated suppression of HSPD1 in the UUO kidney promoted oxidative stress despite miR-382 blockade. (a) MiR-382 abundance determined by real-time PCR measurement of the whole kidney RNA from the obstructed mouse kidneys. (b–e) Protein expression of Bax, HSPD1, and Trx was normalized to GAPDH in the obstructed kidneys treated with anti-miR and siRNA from the experiment above. (f) Representative images of kidney tissue with Sirius red staining and 3-NT immunofluorescence from animals undergoing UUO surgery and receiving a combination of HSPD1 or control siRNA (ureteral delivery) and anti-miR-382 or control anti-miR (intravenous delivery). 3-NT (green) and DAPI (blue) immunofluorescence from control and anti-miR382/HSPD1 siRNA-treated mice are displayed (scale = 20 *μ*m). (g) The relative abundance of collagen was analyzed using Sirius red staining. (h) The positive-stained area of 3-NT was quantitatively measured using a computer-aided image system (IMS) (ShentengIT Co., Shanghai, China) on digitalized images that were transformed from analogue images taken by a video camera (Panasonic, MV-CP410, Japan). Each field was 72,800 *μ*m^2^, magnification ×200. ^∗^*P* < 0.05, ^#^*P* < 0.01 compared with the antiscrambled + control siRNA group; ^▲^*P* < 0.05, ^Δ^*P* < 0.01 compared with the anti-382 + control siRNA group. Trx indicates thioredoxin; 3-NT indicates 3-nitrotyrosine. UUO + anti-Sc + CsiRNA indicates UUO mice treated with scrambled anti-miR and control siRNA; UUO + anti-Sc + HsiRNA indicates UUO mice treated with scrambled anti-miR and HSPD1 siRNA; UUO + anti-382 + CsiRNA indicates UUO mice treated with anti-miR-382 and control siRNA; UUO + anti-382 + HsiRNA indicates UUO mice treated with anti-miR-382 and HSPD1 siRNA (*n* = 4).

In our in vivo animal study, mice that underwent UUO were treated with either anti-miR-382 or antiscrambled control. The ligated kidney was treated locally with HSPD1 siRNA or control siRNA by retrograde injection. By measuring the abundance of miR-382 by real-time PCR, the anti-miR-382 treatment was considered effective (67.86%–70.99% reduction in the level of miR-382, compared with that in the antiscrambled group) (Figure 9(a)). Compared with that in the control siRNA group, protein expression of HSPD1 was significantly suppressed in the HSPD1 siRNA group, while the protein abundance of Bax, a proapoptosis marker, was significantly upregulated after HSPD1 siRNA treatment (Figures 9(b), 9(c), and 9(d)). Fibrosis quantification from Sirius red-stained tissues indicated that anti-miR-382-treated mice with renal knockdown of HSPD1 were not protected from developing tubulointerstitial fibrosis (Figures 9(f) and 9(g)). Meanwhile, the downregulation of Trx or the upregulation of Bax secondary to UUO was partially reversed after anti-miR-382 treatment but not reversed with anti-miR-382 treatment combined with HSPD1 siRNA treatment (Figures 9(b), 9(c), and 9(e)). Fluorescence immunoblotting test showed that the downregulation of 3-NT secondary to anti-miR-382 treatment was blocked by HSPD1 siRNA (Figures 9(f) and 9(h)).

## 4. Discussion

Renal tubulointerstitial fibrosis is a common pathological manifestation of a great variety of chronic kidney diseases (CKD), irrespective of the initial trigger or site of injury. Tubulointerstitial fibrosis (TIF) is also the final common pathway that drives advanced CKD to end-stage renal disease. It has been proven that the extent of TIF is predictive for declining renal function in animal models and humans [15, 16]. Therefore, blocking or even reversing the process of TIF might be one way to halt CKD progression.

The histopathological profiles of TIF is often characterized as excessive extracellular matrix deposition produced by myofibroblasts within the renal interstitium with inflammatory cellular infiltration, tubular atrophy, and capillary loss [17, 18]. The mechanism of epithelial-mesenchymal transition (EMT) was well known, in which injured renal tubular cells transform into mesenchymal cells during renal fibrogenesis [19–22]. Based on the results from our previous study, EMT participated in the pathogenesis of CKD progression in vivo, and in certain conditions, it was reversible, even with the increasing doubts about the existence of EMT in vivo [23]. In this study, a loss of epithelial marker E-cadherin and an increase in extracellular matrix markers (*α*-SMA and Vimentin) were observed in the obstructed kidneys of UUO mice and IgAN patients with TIF.

MicroRNAs (miRNAs) are small, endogenously expressed, noncoding RNA molecules (21–25 nucleotides) founded in plants, animals, and some viruses. MiRNAs regulate gene expression at the posttranslation level through translation inhibition or mRNA degradation, presenting a reciprocal relationship between miRNA and its targeted genes in most cases [24–26]. In our previous study, the abundance of miR-382 in HK2 cells was upregulated with the development of EMT induced by TGF-*β*1. Treatment with anti-miR-382 oligos significantly reversed TGF-*β*1-induced suppression of E-cadherin expression and augmentation of *α*-SMA expression [5]. In the mouse UUO model, we found that the progression of TIF was accompanied with an increased abundance of miR-382 in the obstructed kidneys. Intravenous injection of LNA-anti-miR-382 oligonucleotides (10 mg/kg) significantly alleviated the pathological damage in the obstructed kidneys and suppressed the protein expression of Vimentin and *α*-SMA. Recently, Xu et al. reported miR-382 as an inhibitor of metastasis and EMT in osteosarcoma, which was contradictory to our results from renal tissue [27]. Owing to only a subset of target genes of a miRNA being expressed in a tissue, limited tissue-specific functional roles of the miR-382 can be expected. Therefore, the role of miR-382 could be different in different subtypes of EMT [22].

MiRNAs achieve their biological effects, mostly by repressing translation or decreasing the abundance of their target mRNAs [28, 29]. Kriegel AJ, a member of our research group, had demonstrated that Kalliken5 (KLK5), a (chymo) trypsin-like proteinase that mediates degradation of many extracellular matrix proteins, was a target of miR-382 in the mouse UUO model. Her study revealed that the upregulation of miR-382 contributed to the inner medullary interstitial fibrosis in mice, partially mediated by targeting of KLK5 [30]. Besides ECM genes, a cluster of mitochondrial proteins including HSPD1 was identified as new predicted target genes of miR-382, suggesting that the contribution of miR-382 in the development of TIF could be mediated by pathways of different mechanisms [5].

HSPD1, also called heat shock 60 kDa protein 1, serves as an important molecular chaperone for mitochondrial proteins. HSPD1 was reported to maintain mitochondrial integrity and protect against oxidative stress [8–10]. In vitro experiments revealed that the mRNA expression of HSPD1 was significantly suppressed by pre-miR-382 in HK2 cells, whereas anti-miR-382 treatment significantly reversed the decrease of HSPD1 mRNA. We also found that anti-miR-382 treatment alone could, to some extent, induce HSPD1 expression independently of TGF-*β*1. It is possible that miR-382 may serve as either the downstream of TGF-*β*1 or being in parallel to TGF-*β*1. Besides, the endogenous abundance of miR-382 in HK2 cells should be taken into account. Similarly, HSPD1 expression was upregulated and TIF in the obstructed kidneys was alleviated after LNA-anti-miR-382 (10 mg/kg) treatment. The interaction between miR-382 and HSPD1 was also observed in a clinical study. In situ hybridization, immunohistochemical staining as well as q-PCR measurement revealed the reciprocal relationship between miR-382 and HSPD1 in IgAN patients with TIF. Therefore, HSPD1 serves as one of the target genes of miR-382, which was further verified by site-directed mutagenesis. Since HSPD1 participated in oxidative stress [8, 9], we selected Trx as a marker of antioxidant capacity [31–33] and 3-NT as a marker of oxidative stress [34–36]. Downregulation of HSPD1 was accompanied with a decrease in Trx and an increase in 3-NT, both in UUO mice and in IgAN patients with TIF. Inhibiting the expression of miR-382 with anti-miR-382 led to an increased expression of Trx, as well as a decreased expression of 3-NT in UUO mice. We have also shown that overexpression of HSPD1 is protective against TGF*β*1-induced loss of epithelial characteristics and oxidative stress in vitro. In addition, in vivo study found that the renal protective effects of miR-382 blockade against fibrosis, apoptosis, and redox imbalance of the obstructed kidneys were abolished by renal knockdown of HSPD1, which further proved our hypothesis that miR-382 might contribute to renal interstitial fibrosis by oxidative stress-induced apoptosis secondary to the inhibition of HSPD1.

These results suggested that downregulation of HSPD1 may lead to a decrease in the protective antioxidative capacity within renal tissue. As excessive oxidative stress contribute to the development of TIF, therefore, inhibiting the antioxidative capacity of renal tissue may be one of the important mechanisms in which miR-382 promotes renal tubular interstitial fibrosis.

## 5. Conclusions

In this study, we explored the role of miR-382 in the development of renal fibrosis and its possible mechanism. We proved that miR-382-targeted HSPD1 participated in the setting of renal tubulointerstitial fibrosis. The antioxidative capacity of renal tissue declined when HSPD1 expression was downregulated, which suggested that excessive oxidative stress may be an important mechanism whereby miR-382 participates in renal fibrosis. The current study suggests that upregulation of miR-382, which targets antioxidative stress genes and HSPD1, may partially contribute to the development of renal tubulointerstitial fibrosis. More work is needed to examine, and miR-382 is a potential therapeutic candidate for prevention or treatment of renal tubulointerstitial fibrosis in combination with others.

## Supplementary Material

Supplemental Figure 1: Expression of α-SMA in obstructed murine kidneys. (A) Immunohistochemical analysis for α-SMA abundance in kidney specimens from mice. Treatment with 10mg/kg anti-miR-382 suppressed the expression of α-SMA in obstructed kidneys but not with the dosage of 20mg/kg. (B) Relative mRNA expression of α-SMA in obstructed murine kidneys. (C) Quantification of α-SMA staining. Values labeled with (∗) are compared with the sham group, P<0.05. Values labeled with (#) are compared with 10 mg/kg anti-382 group, P<0.05. “UUO” indicates unilateral ureteral obstruction. “NC” indicates negative control. (n=4). Supplemental Figure 2: Expression of Vimentin in obstructed murine kidneys. (A) Immunohistochemical analysis for Vimentin abundance in kidney specimens from mice. Treatment with 10mg/kg dosage of anti-miR-382 suppressed the expression of Vimentin in UUO mice, but not with the dosage of 20mg/kg. (B) Relative mRNA expression of Vimentin. (C) Quantification of Vimentin staining. Values labeled with (∗) are compared with the sham group, P<0.05. Values labeled with (#) are compared with 10 mg/kg anti-382 group, P<0.05. “UUO” indicates unilateral ureteral obstruction. “NC” indicates negative control. (n=4)

## Figures and Tables

**Table 1:** Primers used in real-time PCR.

Target	Gene symbol	Sequence
Heat shock protein 1 (chaperonin) (mouse)	Hspd1	Sense:5- TTGCTAATGCTCATCGGAAG -3
		Antisense:5- AGCGTGCTTAGAGCTTCTCC
Heat shock protein family D (Hsp60) member 1 (human)	HSPD1	Sense:5- AAATTGCACAGGTTGCTACG -3
		Antisense:5- TGATGACACCCTTTCTTCCA -3
E-cadherin (human)	CDH1	Sense:5- TGCCAAGTGGGTGGTATAGAGG -3
		Antisense:5- CAGTGGGATGGTGGGTGTAAGA -3
Glyceraldehyde-3-phosphate dehydrogenase (human)	GAPDH	Sense:5- CATCTTCTTTTGCGTCGCCA -3
		Antisense:5- TTAAAAGCAGCCCTGGTGACC -3
Glyceraldehyde-3-phosphate dehydrogenase (mouse)	Gapdh	Sense:5- CGGAGTCAACGGATTTGGTCGTAT -3′
		Antisense:5- AGCCTTCTCCATGGTGGTGAAGAC
*α*-Smooth muscle actin (human)	ACTA2	Sense:5- CTGTTCCAGCCATCCTTCAT -3
		Antisense:5- TCATGATGCTGTTGTAGGTGGT -3
*α*-Smooth muscle actin (mouse)	Acta2	Sense:5- CTGACAGAGGCACCACTGAA -3
		Antisense:5- CATCTCCAGAGTCCAGCACA -3
Vimentin (human)	VIM	Sense: 5- TACAGGAAGCTGCTGGAAGG -3
		Antisense: 5- ACCAGAGGGAGTGAATCCAG -3
Vimentin (mouse)	Vim-	Sense:5- TGAAGGAAGATGGCTCGT -3
		Antisense:5- TCCAGCAGCTTCCTGTAGGT -3
Thioredoxin (human)	TXN	Sense:5′- TTGGACGCTGCAGGTGATAAAC -3′
		Antisense:5′- GGCATGCATTTGACTTCACACTC -3′
