# Supplementary material for: miR-382 Contributes to Renal Tubulointerstitial Fibrosis by Downregulating HSPD1
Source: Oxid Med Cell Longev. 2017 Jun 7;2017:4708516. doi: 10.1155/2017/4708516 (PMC5478870; doi:10.1155/2017/4708516)
Supplement: Supplementary file 1 — Supplemental Figure 1: Expression of α-SMA in obstructed murine kidneys. (A) Immunohistochemical analysis for α-SMA abundance in kidney specimens from mice. Treatment with 10mg/kg anti-miR-382 suppressed the expression of α-SMA in obstructed kidneys but not with the dosage of 20mg/kg. (B) Relative mRNA expression of α-SMA in obstructed murine kidneys. (C) Quantification of α-SMA staining. Values labeled with (∗) are compared with the sham group, P<0.05. Values labeled with (#) are compared with 10 mg/kg anti-382 group, P<0.05. “UUO” indicates unilateral ureteral obstruction. “NC” indicates negative control. (n=4). Supplemental Figure 2: Expression of Vimentin in obstructed murine kidneys. (A) Immunohistochemical analysis for Vimentin abundance in kidney specimens from mice. Treatment with 10mg/kg dosage of anti-miR-382 suppressed the expression of Vimentin in UUO mice, but not with the dosage of 20mg/kg. (B) Relative mRNA expression of Vimentin. (C) Quantification of Vimentin staining. Values labeled with (∗) are compared with the sham group, P<0.05. Values labeled with (#) are compared with 10 mg/kg anti-382 group, P<0.05. “UUO” indicates unilateral ureteral obstruction. “NC” indicates negative control. (n=4) [file 4708516.f1.docx]

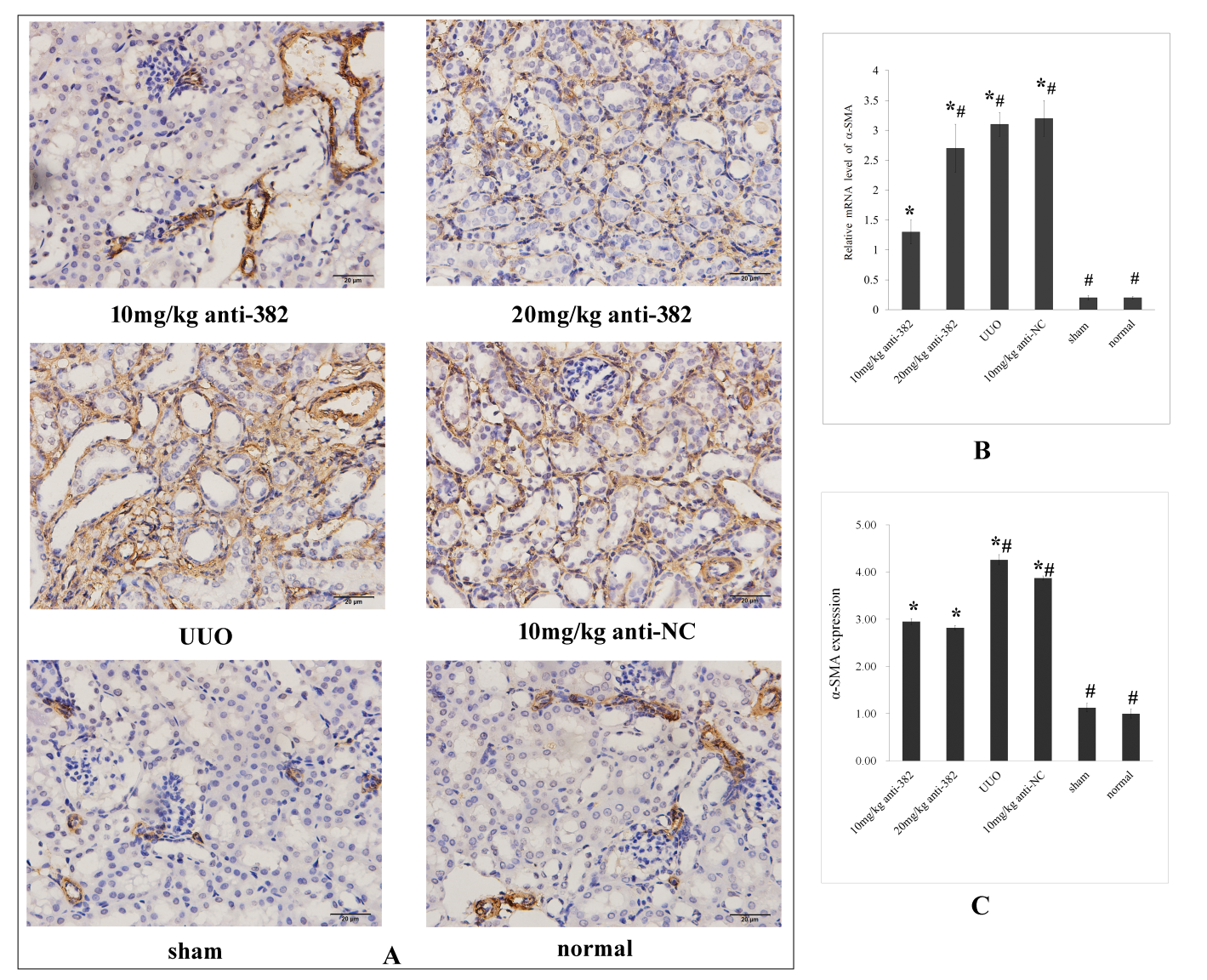


Supplemental Figure 1: Expression of α-SMA in obstructed murine kidneys. (A) Immunohistochemical analysis for α-SMA abundance in kidney specimens from mice. Treatment with 10mg/kg anti-miR-382 suppressed the expression of α-SMA in obstructed kidneys but not with the dosage of 20mg/kg. (B) Relative mRNA expression of α-SMA in obstructed murine kidneys. (C) Quantification of α-SMA staining. Values labeled with (*) are compared with the sham group, P<0.05. Values labeled with (#) are compared with 10 mg/kg anti-382 group，P<0.05. “UUO” indicates unilateral ureteral obstruction. “NC” indicates negative control. (n=4)


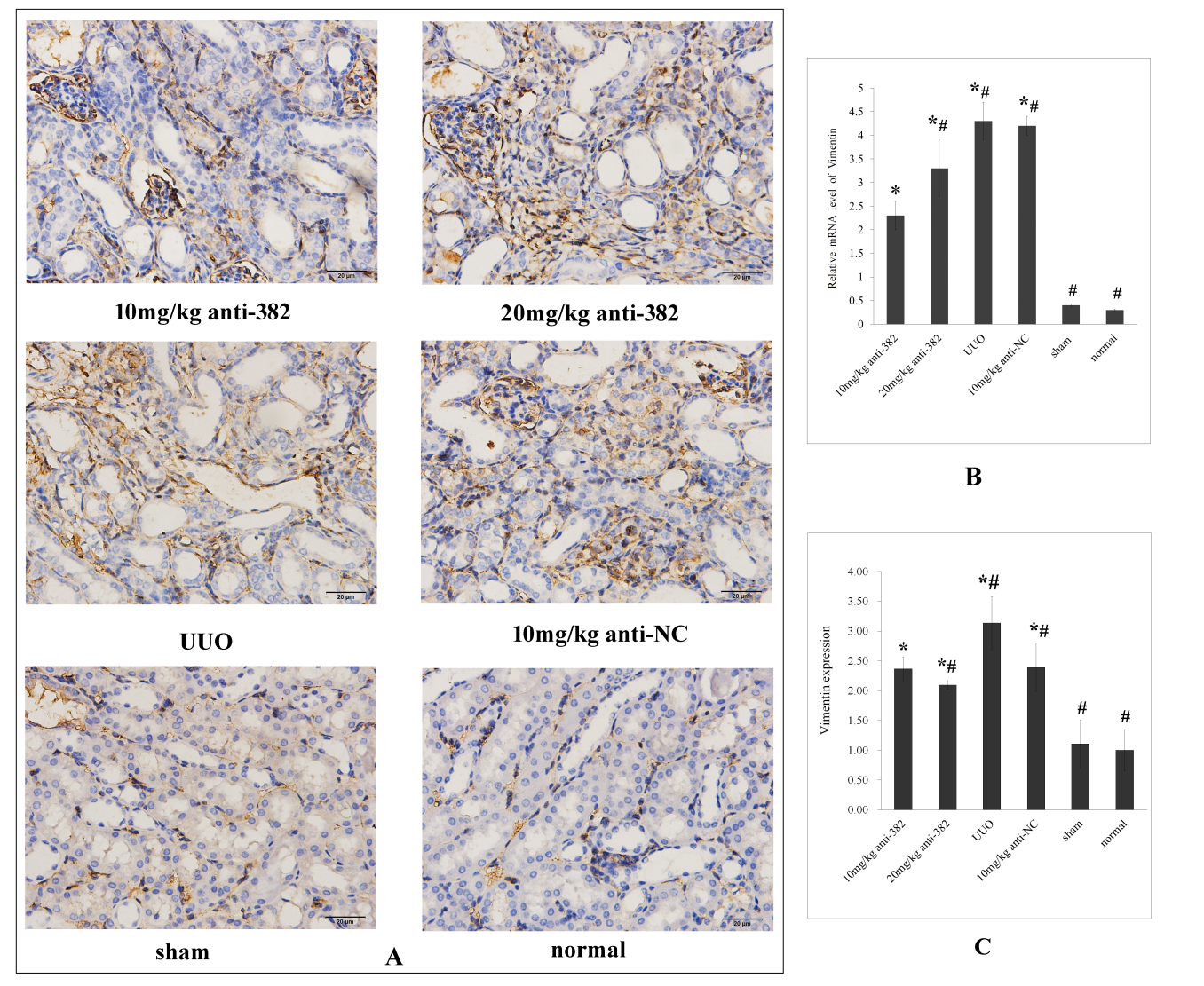


Supplemental Figure 2: Expression of Vimentin in obstructed murine kidneys. (A) Immunohistochemical analysis for Vimentin abundance in kidney specimens from mice. Treatment with 10mg/kg dosage of anti-miR-382 suppressed the expression of Vimentin in UUO mice, but not with the dosage of 20mg/kg. (B) Relative mRNA expression of Vimentin. (C) Quantification of Vimentin staining. Values labeled with (*) are compared with the sham group, P<0.05. Values labeled with (#) are compared with 10 mg/kg anti-382 group, P<0.05. “UUO” indicates unilateral ureteral obstruction. “NC” indicates negative control. (n=4)
